# Elevated prothrombin time/international normalized ratio associated with concurrent administration of regorafenib and warfarin in a patient with advanced colorectal cancer

**DOI:** 10.1186/s40780-016-0050-y

**Published:** 2016-07-08

**Authors:** Hironori Kitade, Azusa Hiromasa-Yamasaki, Kengo Hokkoku, Mitsue Mori, Michio Watanabe, Masuo Nakai, Seiji Yano

**Affiliations:** Department of Pharmacy, Houju Memorial Hospital, 11-71, Midorigaoka, Nomi-shi, Ishikawa 923-1226 Japan; Department of Gastroenterology, Houju Memorial Hospital, 11-71, Midorigaoka, Nomi-shi, Ishikawa 923-1226 Japan; Outpatient Cancer Chemotherapy Center, Houju Memorial Hospital, 11-71, Midorigaoka, Nomi-shi, Ishikawa 923-1226 Japan; Department of Medical Oncology, Cancer Research Institute, Kanazawa University, 13-1 Takara-machi, Kanazawa, Ishikawa 920-0934 Japan

**Keywords:** Regorafenib, Warfarin, PT/INR, Colorectal cancer

## Abstract

**Background:**

Regorafenib and its metabolites may inhibit the activities of several CYP or UDP-glucuronosyltransferase isoforms, including that of CYP2C9. Therefore, pharmacological agents that are CYP2C9 substrates may show elevated circulating levels and enhanced drug efficacy when concurrently used with regorafenib. Previous studies showed that the area under the plasma concentration-time curve of warfarin, which is the substrate for CYP2C9, increased upon co-administration of regorafenib. However, there are no reports indicating that the anticoagulant effects of warfarin increased upon co-administration of regorafenib.

**Case presentation:**

We report a case of a 76-year-old man with liver metastasis of colon cancer. He was treated with regorafenib at a dosage of 120 mg daily on days 1 to 21 every 4 weeks as a third-line therapy. He had a history of acute myocardial infarction and had taken 2 mg warfarin daily. Three weeks after the treatment began, PT/INR values markedly increased, although there was no hemorrhage. Administration of regorafenib and warfarin was discontinued, and then PT/INR rapidly decreased. Warfarin administration was restarted (0.5 mg daily) and the dose was increased up to 1.5 mg daily. The patient’s PT/INR values exhibited a tendency to increase when concurrently used with regorafenib, the dose of which was reduced to 80 mg daily on days 1 to 14 every 3 weeks at a physician's discretion.

**Conclusions:**

The clinical course of this patient suggested that PT/INR might increase during concurrent use of warfarin and regorafenib. Therefore, PT/INR should be periodically monitored during the concurrent use of warfarin and regorafenib.

## Background

Regorafenib is an oral multikinase inhibitor, which has shown antitumor activity in patients with advanced colorectal cancer or gastrointestinal stromal tumors who have previously received standard treatment and discontinued it owing to disease progression or side effects [1, 2]. Regorafenib is mainly metabolized to M-2 and M-5 by cytochrome P-450 (CYP) 3A4, both of which have pharmacological activity similar to that of regorafenib [3]. In addition, results from *in vitro* analysis showed that regorafenib and its metabolites might inhibit the activity of several CYPs such as CYP2C9 and CYP2C8 or UDP-glucuronosyltransferase isoforms [3].

The plasma concentration of warfarin, the substrate for CYP2C9, could be increased with the concurrent use of regorafenib, which leads to enhance warfarin’s anticoagulant effects. Pharmacokinetic studies have shown that the area under the plasma concentration-time curve of warfarin increases with concurrent administration of regorafenib [3]. However, there are no reports indicating that regorafenib interacts with warfarin and leads to increased anticoagulant effects. Here, we report the case of a patient with advanced colon cancer, who experienced elevated prothrombin time/international normalized ratio (PT/INR) with concurrent use of regorafenib and warfarin.

## Case presentation

**Fig. 1:**
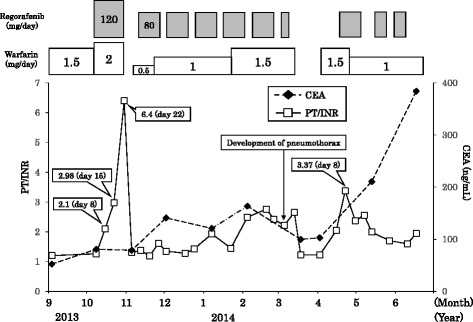
Prothrombin time/international normalized ratio (PT/INR) values and serum carcinoembryonic antigen (CEA) levels during regorafenib treatment. Only an important points are mentioned PT/INR values (day on course) after co-administration of regorafenib

A 76-year-old man with stage IV colon cancer metastasis underwent resection of part of the colon and cholecystectomy. He had a history of hypothyroidism and acute myocardial infarction. He did not drink alcohol but had a history of smoking a pack of cigarettes daily for about 40 years. He had taken medicines, including levothyroxine (75 μg daily), spironolactone (25 mg daily), nicorandil (10 mg daily), pravastatin (5 mg daily), allopurinol (200 mg daily), and warfarin (2 mg daily). After surgery, treatment was started with modified FOLFOX-6 (mFOLFOX6, 85 mg/m^2^ oxaliplatin, 200 mg/m^2^ leucovorin, 400 mg/m^2^ 5-FU bolus on day 1 and 2400 mg/m^2^ 5-FU over 46 h every 2 weeks) in July 2012 to liver metastasis. His PT/INR value was controlled around 2.0 before receiving mFOLFOX6 and did not increase when this treatment was given. He was received mFOLFOX6 for 6 months, but his carcinoembryonic antigen (CEA) level increased, and computed tomography (CT) showed progression of liver metastasis. Then, treatment with irinotecan (CPT-11) plus S-1 was initiated (CPT-11 125 mg/m^2^ on days 1 and 15 and S-1 40 mg twice daily for 2 weeks, repeated every 4 weeks). His PT/INR value slightly increased to 2.98 from 2.31 2 weeks after CPT-11 plus S-1 was administered. Then the dose of warfarin was reduced to 1 mg and PT/INR value decreased to around 2.0. However, after 3 cycles of treatment with CPT-11 plus S-1, he developed interstitial pneumonia and discontinued this therapy, leading to decreased PT/INR value around 1.3. Furthermore, the interstitial pneumonia improved but a CT scan indicated that his hepatic metastasis had progressed. Then, he began a course of treatment with regorafenib at a dosage of 120 mg daily on days 1 to 21 every 4 weeks in October 2013. The warfarin dose was increased up to 1.5 mg one month before initiation of treatment with regorafenib and up to 2 mg again when regorafenib was given concurrently (Fig. 1).

Although the patient’s PT/INR value was 1.26 before initiation of treatment with regorafenib, this value significantly increased to 2.1 on day 8, 2.98 on day 15, and 6.4 on day 22 (Fig. 1). Regorafenib and warfarin treatments were discontinued, and his PT/INR value decreased to 1.31 within 1 week after the discontinuation. Then, warfarin was reduced by 0.5 mg daily and was restarted. As he had developed grade 2 hand-foot syndrome during the first cycle of treatment, regorafenib was also reduced by 80 mg daily on days 1 to 14 every 3 weeks at a physician's discretion and was restarted.

The warfarin dose was increased up to 1 mg daily after 2 weeks and up to 1.5 mg daily after 2 months, respectively. Although his PT/INR value increased gradually with an increasing dose of warfarin, further elevation was not seen with the concomitant use of regorafenib. Additionally, laboratory findings showed that liver functions including alanine transaminase and aspartate transaminase serum levels were normal, although the liver metastasis showed slight progression on a CT scan (data not shown).

In March 2014, regorafenib and warfarin treatment were discontinued again because the patient developed a pneumothorax. One month later, the pneumothorax healed and the patient was restarted on a course of warfarin (1.5 mg daily) prior to regorafenib. Two weeks later, regorafenib treatment was also restarted at the same treatment schedule and dose, at which time his PT/INR value was 2.04. His PT/INR value increased to 3.37 again on day 8 after restarting treatment with regorafenib (Fig. 1). He was then discharged with the treatment of 1 mg warfarin daily. The PT/INR value reduced to 2.37 one week after the reduction of warfarin treatment. Then, the PT/INR value did not increase during the concomitant use of regorafenib. However, his CEA level increased, and his hepatic metastasis showed progression on a CT scan. Then, treatment with regorafenib was discontinued and the patient was treated with trifluridine and tipiracil hydrochloride (Lonsurf®). PT/INR value did not increase when the salvage treatment with Lonsurf® was given (data not shown).

This case report was approved by the ethics review committee of the Houju Memorial Hospital (Ishikawa, Japan) (No. 16–5).

## Discussion

This is the first report of a patient with advanced colon cancer who had a significant increase in the PT/INR when administered warfarin concomitantly with regorafenib. Although the warfarin dose was increased up to 2 mg when regorafenib was administered simultaneously, the patient did not demonstrate a marked increase in PT/INR with a daily 2 mg dose of warfarin before receiving chemotherapy. Therefore, we suggest that this event was not caused only by an increase in the dose of warfarin.

There is a possibility that impaired hepatic function due to tumor progression results in the elevation of PT/INR, but no deterioration of liver function was observed, while hepatic metastasis had slightly progressed during the regorafenib treatment. In addition, it is suggested that regorafenib itself may increase the PT/INR value directly via inhibition of vascular endothelial growth factor signaling, which is related to the induction of angiogenesis. Indeed, a previous report showed that INR value increased in 120 out of 500 patients (24 %) who were treated with regorafenib [3]. However, in this case, the PT/INR value did not increase when a low dose of warfarin (0.5–1 mg daily) was administered, whereas its value increased at the warfarin dose was increased up to 1.5–2 mg, while the dose of regorafenib was same (Fig. 1). Therefore, we speculate that PT/INR increase in this case was not caused by direct action of regorafenib but by drug interaction between regorafenib and warfarin.

We assessed this event using the Naranjo adverse drug reaction probability scale, which indicated a probable relationship (score of 6) between regorafenib use and the elevated PT/INR value [4]. The Naranjo scale was designed to evaluate single drug adverse events, not drug interactions. We also assessed the causation of this adverse reaction using the drug interaction probability scale score for potential drug interactions in the patient, which indicated a probable likelihood of an interaction (score of 7) [5].

Warfarin is highly bound (99 %) to human plasma proteins [6]. *In vitro* protein binding of regorafenib, M-2, and M-5 to human plasma proteins is also high at 99.5, 99.8, and 99.95 %, respectively [3]. There were no change in serum albumin level during concomitant use of regorafenib and warfarin in our case (data not shown). Although we could not measure the plasma free levels of warfarin or regorafenib and its metabolite, it is possible that the concentration of plasma-free warfarin will be elevated and lead to increased anticoagulant effects via competition for protein binding sites when these two drugs are used concurrently. However, it is unknown that which metabolite is most contribute to the displacement warfarin from plasma proteins.

Warfarin is metabolized via several CYPs, including CYP3A4, with involvement of CYP1A1, CYP1A2, CYP2C8, CYP2C9, CYP2C18 and CYP2C19 and CYP3A4 [7–10]. S-warfarin, which exhibits potent anticoagulant activity, is metabolized primarily by CYP2C9 [7]. Regorafenib and its metabolite M-2 inhibit CYP2C9 [3]. IC_50_ values for CYP2C9 inhibition by regorafenib and M-2 are 2.7 and 6.1 μM, respectively [11]. Furthermore, pharmacokinetic analysis from a phase I study also revealed that after oral administration of 160 mg or 120 mg regorafenib for 3 weeks, the plasma C_max_ value in its steady state of regorafenib is slightly higher than that of M-2 [12, 13]. In this case, the patient was not administered regorafenib concomitantly with a potent inducer or inhibitor of CYP3A4, which is the main enzyme involved in metabolism of regorafenib. Therefore, although we could not measure plasma C_max_ values of regorafenib and M-2 at the steady state, we speculate that the steady state C_max_ value of regorafenib in this case could also be larger than that of M-2. Therefore, we speculate that regorafenib may have more potent inhibitory effect on CYP2C9 than M-2, considering IC_50_ and steady state C_max_ values of them.

In this case, a significant elevation of PT/INR occurred 3 weeks after regorafenib co-administration. Goto et al. reported that hematuria and a marked increase in PT/INR occurred after administering warfarin for about 1 month [14]. In this report, PT/INR increased due to CYP2C9 variants because the patient had the CYP2C9*3/*3 allele. Indeed, CYP2C9 genotypes are closely associated with warfarin dose adjustment [15]. Of the more than 30 identified CYP2C9 variants, the CYP2C9*3 allele is associated with reduced enzyme activity and requires lower maintenance doses of warfarin [16]. Obayashi et al. reported that the mean daily dose of warfarin was lower in subjects with CYP2C9*1/*3 (1.86 ± 0.80 mg daily) than in subjects with CYP2C9*1/*1 (3.36 ± 1.43 mg daily) in a Japanese population [17]. A similar result was reported by Ohno et al. for differences in the mean maintenance dose of warfarin in Japanese patients with CYP2C9 variants [18]. Although we did not analyze the CYP2C9 variant in our case, the PT/INR value was controlled with 2 mg warfarin daily before receiving chemotherapy. Thus, we speculate that the patient might have the CYP2C9*3 allele, which might contribute to an increase in the PT/INR after the concomitant administration of regorafenib.

Moreover, it has been reported that other oral multikinase inhibitors, such as imatinib, sorafenib, and vemurafenib, when used concurrently with warfarin, may also have the ability to increase circulating levels of warfarin and potentiating its anticoagulant effects [19–21]. Therefore, when treatment with such oral multikinase inhibitors is administered to patients who receive warfarin daily, switching from warfarin to other novel oral anticoagulants such as rivaroxaban and apixaban based on drug interaction should be considered.

## Conclusions

The clinical course of this patient suggested that the PT/INR increases during the concurrent use of warfarin and regorafenib. Therefore, PT/INR should be periodically monitored during the concurrent use of warfarin and regorafenib.

## Abbreviations

CEA, carcinoembryonic antigen; CPT-11, irinotecan; CT, computed tomography; CYP, cytochrome P-450; mFOLFOX6, modified FOLFOX-6; PT/INR, prothrombin time/international normalized ratio
